# Composition, diversity and bioactivity of culturable bacterial endophytes in mountain-cultivated ginseng in Korea

**DOI:** 10.1038/s41598-017-10280-7

**Published:** 2017-08-30

**Authors:** MD. Emran Khan Chowdhury, Junhyun Jeon, Soon Ok Rim, Young-Hwan Park, Seung Kyu Lee, Hanhong Bae

**Affiliations:** 1 Department of Biotechnology, Yeungnam University, Gyeongsan, Gyeongbook, 38541 Republic of Korea; 2 Division of Forest Diseases & Insect Pests, Korea Forest Research Institute, Seoul, 02455 Republic of Korea

## Abstract

Plants harbor diverse communities of bacterial species in their internal compartments. Here we isolated and identified bacterial endophytes from mountain-cultivated ginseng (MCG, *Panax ginseng* Meyer) to make working collection of endophytes and exploit their potentially beneficial properties toward plants and human being. A total of 1,886 bacteria were isolated from root, stem and leaf of MCGs grown in 24 different sites across the nation, using culture-dependent approach. Sequencing of 16S rDNA allowed us to classify them into 252 distinct groups. Taxonomic binning of them resulted in 117 operational taxonomic units (OTUs). Analysis of diversity indices across sampling sites and tissues suggested that composition of bacterial endophyte community within ginseng could differ substantially from one site to the next as well as from one host compartment to another. Assessment of 252 bacterial isolates for their beneficial traits to host plants showed that some bacteria possesses the ability to promote plant growth and produce ß-glucosidase, indicating their potential roles in plant growth promotion and bio-transformation. Taken together, our work provides not only valuable resources for utilization of bacterial endophytes in ginseng but also insights into bacterial communities inside a plant of medicinal importance.

## Introduction

Modern agriculture depends heavily on use of unsustainable level of agrochemicals such as fertilizers and pesticides, which have repercussions for environment and human health. An emerging alternative to such practices is utilization of plant microbiomes that are linked to improved plant health and productivity^1–5^. Among diverse microorganisms constituting plant microbiome, endophytes are microbes that spend entire or part of their life cycle inside plants (inter- or intra-cellular spaces) without damaging plant tissues or inducing defense responses^3^. Although exact roles and nature of plant-endophyte interactions are not fully understood, it has been well established that some of these interactions are beneficial to plants^4,6^. For example, it was shown that bacterial endophytes can take part in the production of bioactive compounds found in their hosts, and have potential to produce new drugs, plant hormones, and novel natural products^1,7–9^. Bacterial endophytes were also shown to promote plant growth and suppress plant diseases^10,11^. Such metabolic and agronomical potential of endophytes spurred a great deal of scientific endeavors in recent years^3^.

The first step toward full-fledged use of endophytes in agriculture is to catalogue the list of microbes showing endophytic life style associated with a particular species of host plant. Currently, metagenomic studies employing next-generation sequencing technology are being increasingly used to discover and understand new bacterial endophytes of important food crops^12–14^. These studies have led to identification of endophytic bacteria belonging to diverse taxa including *Proteobacteria*, *Bacteriodetes*, *Firmicutes*, and *Actinobacteria*. To date, a lot of works on plant microbiome highlighted that structure of microbial communities associated with plants is shaped by biotic factors including host species, genotype of a given species, and host age as well as many abiotic factors influencing physiology of the host plants^3,15,16^. However, validating and characterizing their endophytic lifestye and properties benefiting plants and humans are significantly limited by our ability to isolate and culture them, although it is likely that microbes in endosphere and rhizosphere are more amenable to culture than soil counterparts^17^.

Ginseng (*Panax ginseng* Meyer) belongs to the family *Araliaceae* and has been recognized as a traditional medicinal plant of the highest clinical value for more than a thousand years^18,19^. The most pharmaceutically active ingredients in ginseng are a complex mixture of more than 180 different triterpenoid saponins commonly known as ginsenosides^18^. Ginsenosides are distributed in different parts of ginseng plant - root, leaf, and berry - with distinct ginsenoside profiles. The demand for ginseng roots and their extracts as nutraceuticals has been increasing due to a number of pharmacologically active ingredients including ginsenosides, oleanic acids, phenolic compounds and volatiles^18^. However, successive cultivation in the same soil for a long time is known to be associated with changes in physicochemical properties of the soil, which in turn, frequently provide settings conducive to diseases by soil-borne pathogens, leading to devastating yield losses.

In this study, we set out to isolate and characterize bacterial endophytes associated with ginseng plants using culture-dependent approach. Our primary objective is to build a working collection of ginseng-associated endophytes that can be used as resources for exploring possible application of endophytes and endophyte-derived compounds to agricultural systems. Here we used mountain-cultivated ginseng for isolation of endophytes instead of field-grown ginseng, since intensive and long-term use of pesticides for protection of field-grown ginseng plants may greatly impact composition and structure of otherwise diverse endophytes inside them. Following isolation of endophytes, we evaluated site-wise and tissue-wise diversity of bacterial endophytes as well as their potential properties regarding plant growth promotion and biotransformation.

## Results

### Isolation and identification of bacterial endophytes

**Figure 1:**
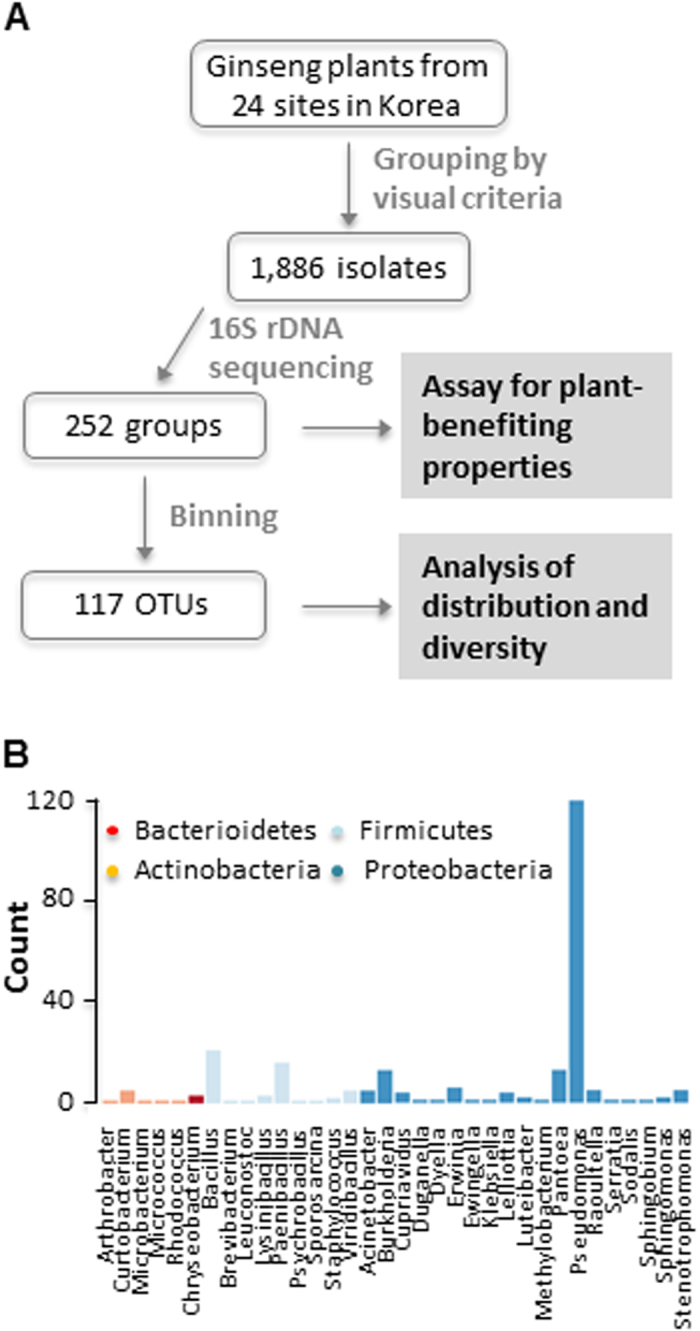
Isolation and identification of culturable bacterial endophytes from mountain-cultivated ginseng in Korea. (**A**) Schematic diagram depicting workflow during isolation and identification of endophytes from mountain-cultivated ginseng plants. (**B**) Bar plot showing the number of OTUs belonging to each genus (among the total of 35 genus recovered in our study). Different colors indicate the phyla to which each genus belongs.

A total of 1,886 culturable bacterial endophytes were initially isolated based on on-plate characteristics from 24 different sites and 3 different tissues of the ginseng plants to describe the culturable bacterial endophytes in MCGs and their potential properties (Fig. 1A and Supplementary Fig. S1). Based on 16S rDNA sequencing, isolates were classified into 252 distinctive groups. Potential plant-benefiting properties were assessed for these 252 groups later. Taxonomic binning of these 252 isolates resulted in a total of 117 OTUs encompassing 35 different genera (Fig. 1). Most of the OTUs were either known or closely related to common plant-associated endophytic and soil bacteria. Majority of them showed >99% similarity to the reference strains (Supplementary Table S1).

The phylogenetic analysis of 117 OTUs showed a rather limited range of taxonomical distribution (Fig. 1B). All the representative isolates were clustered into 4 phyla: *Proteobacteria*, *Firmicutes*, *Bacteroidetes* and *Actinobacteria* (Supplementary Fig. S2). *Proteobacteria* were predominant, accounting for ~63% of total OTUs. *Firmicutes* were the second largest phylum (30.1%). *Actinobacteria* and *Bacteroidetes* were about 4.3% and 1.7%, respectively. Among all the genera, *Pseudomonas*, which belongs to *Gamma-proteobacteria*, was the most abundant genus, comprising 48% of total OTUs (Fig. 1B). Twenty genera out of 35 showed frequencies that are less than 1%. Except *Pseudomonas*, *Bacillu*s, *Burkholderia*, *Pantoea* and *Paenibacillus* were the only genera showing frequencies higher than 5%.

### Diversity and distribution of culturable bacterial endophytes across sites

**Figure 2:**
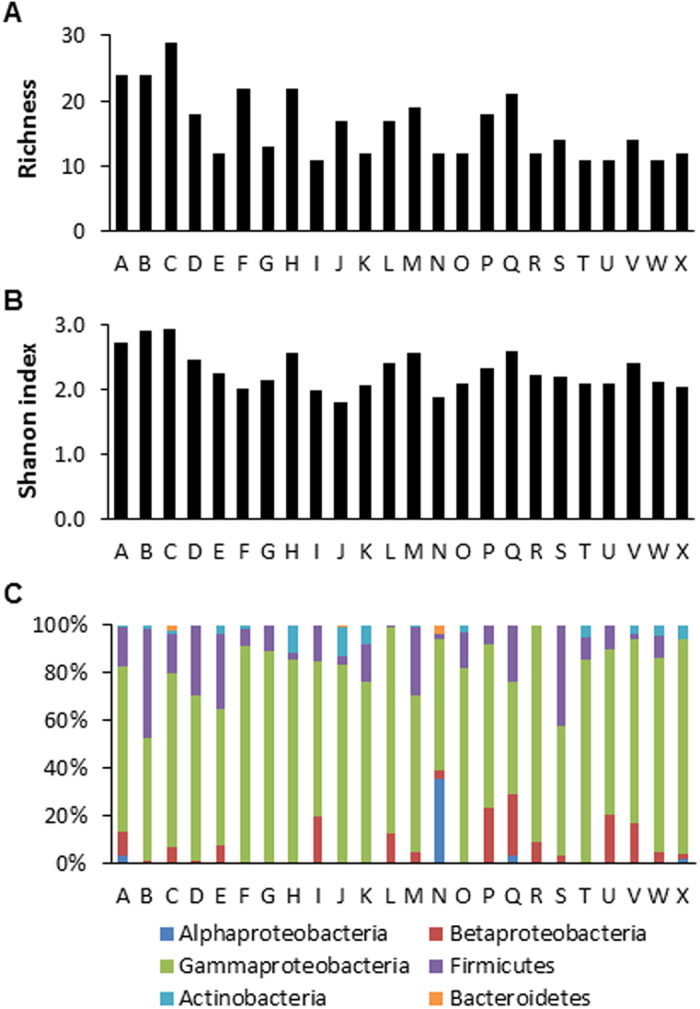
Distribution and diversity of cultural bacterial endophytes in mountain-cultivated ginseng. (**A**) Species richness across the sampling sites. (**B**) Shannon’s (*H’*) index across the sampling sites. (**C**) Relative abundance of OTUs at (sub) phylum level across sampling sites.

Our culturable collection of bacterial endophytes was further analyzed in order to gain insight into bacterial diversity in MCGs. Our analysis showed that the level of bacterial diversity differed significantly among the sites. Species richness was highest in region C (29 OTUs), followed by regions A and B (25 and 24 OTUs, respectively), and lowest in I, T, and U regions (11 OTUs for all three) (Fig. 2A). Alpha diversity as measured by the Shannon (*H’*) index was highest in region C (*H’* = 2.929), followed by regions B, A, H, M and Q (*H’* > 2.5), and the lowest alpha diversity was observed in region J (*H’* = 1.798) (Fig. 2B). In addition, relative abundance of each phylum (or sub-phylum in *Proteobacteria*) showed large variation across the sites, although OTUs belonging to *Gamma-proteobacteria* were the most abundant in all sampling sites (Fig. 2C). Notably, *Alpha-proteobacteria* appeared to be highly abundant in the site M, and *Bacteroidetes* was found only in sites C, J, and M.

Given the observed diversity across the sampling sites, we then asked if there are factors that could possibly explain variations in composition and distribution of culturable bacterial endophytes in MCGs. The factors that we examined are geographic proximity among sampling sites, mean annual temperature and precipitation, altitude, and various edaphic factors such as soil pH and phosphate availability (Supplementary Fig. S1 and Supplementary Table S2). Our initial hypotheses were two-folds: i) Sampling sites that are geographically close to each other would have similar community profiling; ii) As a corollary to the first hypothesis, at least some climate and edaphic factors should have good correlation to variation in endophyte diversity. On contrary to the hypotheses, however, degree of proximity among sampling sites had no or little correlation to variation in endophyte community diversity (Supplementary Figs S1 and S3). None of the individual climatic and edaphic factors were able to account for the observed changes in community diversity across the sites (Supplementary Figs S4 and S5).

### Association of bacterial endophytes in MCGs

**Figure 3:**
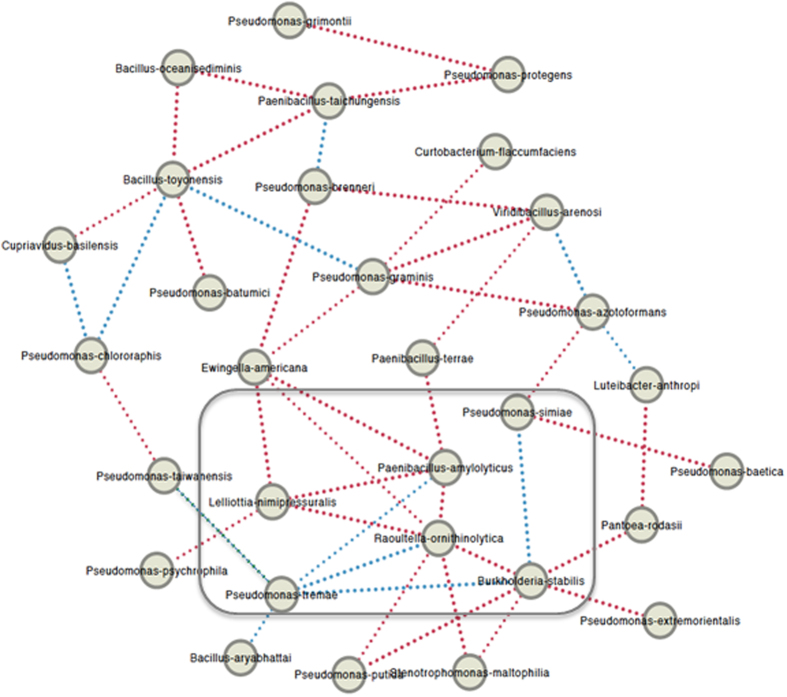
Association network constructed from count data of OTUs across sampling sites using CoNet application in Cytoscape. Individual circles represent different OTUs. Red and blue edges (dotted lines connecting nodes) indicate ‘mutual exclusion’ and ‘co-occurrence’ relationships, respectively. Gray box indicates nodes with the highest degree and their immediate neighbors.

Mere compilation of microbes that exist in a plant, though helpful, does not provide much information on how microbes are related and how important they might be^20,21^. To delve into the detailed nature of relationships among our culturable bacterial endophytes, we constructed an association network of them, based on abundance of individual OTUs across the sample sites (Fig. 3)^22^. Since composition of bacterial endophytes in MCG varied significantly from site to site, most of the relationships among them were mutual exclusion, in which occurrences of one species negatively correlates with another, although some of the pairs exhibited co-occurrences. In this network, *Raoultella ornithinolytica* and *Burkholderia stabilis* were the nodes of the highest degree (see the nodes inside gray box in Fig. 3). *R*. *ornithinolytica* tended to co-occur only with *Pseudomonas tremae*, while *B*. *stabilis* did with *Pseudomonas simiae* as well as *P*. *tremae*. However, *R*. *ornithinolytica* and *B*. *stabilis* had a tendency to avoid each other (mutual exclusion), suggesting possible competition for common resources. Notably, all the 5 immediate neighbor nodes (first degree nodes) of *P*. *tremae* showed co-occurrence relationship. This may suggest either symbiosis between *P*. *tremae* and others, or important roles of *P*. *tremae* in establishing favorable environmental conditions for other endophytes such as *R*. *ornithinolytica* and *B*. *stabilis*.

### Diversity and distribution of bacterial endophytes in tissues

**Figure 4:**
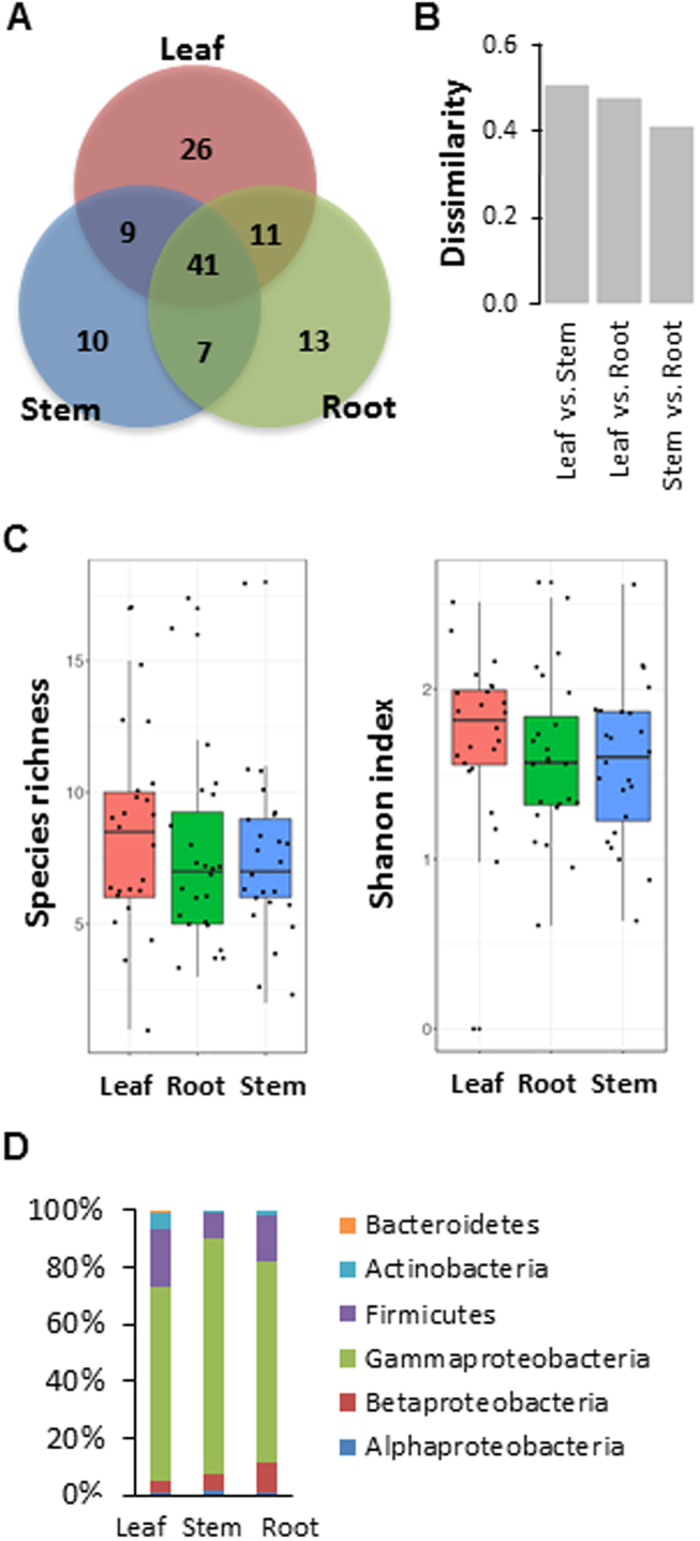
Distribution and diversity of culturable bacterial endophytes in different tissues of mountain-cultivated ginseng. (**A**) Venn diagram showing overlaps of endophytes recovered from root, stem and leaf tissues. (**B**) Dissimilarity between tissues based on composition of bacterial endophytes in each tissue. Dissimilarity was calculated using Bray-Curtis dissimilarity measure. (**C**) Boxplots showing species richness (left panel) and Shannon’s index (right panel) in root, stem and leaf tissues. (**D**) Relative abundance of bacterial endophytes in different tissues at (sub) phylum level.

Next, we examined distribution and diversity of culturable bacterial endophytes in different tissues. Overall, total species richness was highest in leaf tissue (87 OTUs) and the lowest in stem (67 OTUs) (Fig. 4A). Some endophytes were found only in one tissue, while 35% of all the endophytes identified in our work, which corresponds to 41 OTUs, were found in all three tissues. Pairwise comparison of three tissues using count data of individual OTUs showed that stem and root have similar composition of bacterial endophytes (Fig. 4B). Interestingly, bacterial composition of leaf tissue was more similar to root than to stem.

Even though overall species richness was highest in leaf, analysis of species richness among tissues across 24 sampling sites revealed no statistically significant differences (Fig. 4C left panel). Likewise, diversity of endophytes measured by Shannon (*H’*) index was not considerably different among tissues (not statistically significant difference) probably due to high degree of site-by-site variation. (Fig. 4C right panel). Despite lack of statistically significant differences among tissues, relative abundance of bacterial endophytes in three tissues showed note-worthy differences among them in addition to over-abundance of *Proteobacteria* at the phylum level. We found that *Bacteroidetes* were present only in leaf tissue and that *Firmicutes* appeared to be more abundant in leaf and root than in stem tissue (Fig. 4D).

### Evaluation of bacterial endophytes for their potential properties

**Figure 5:**
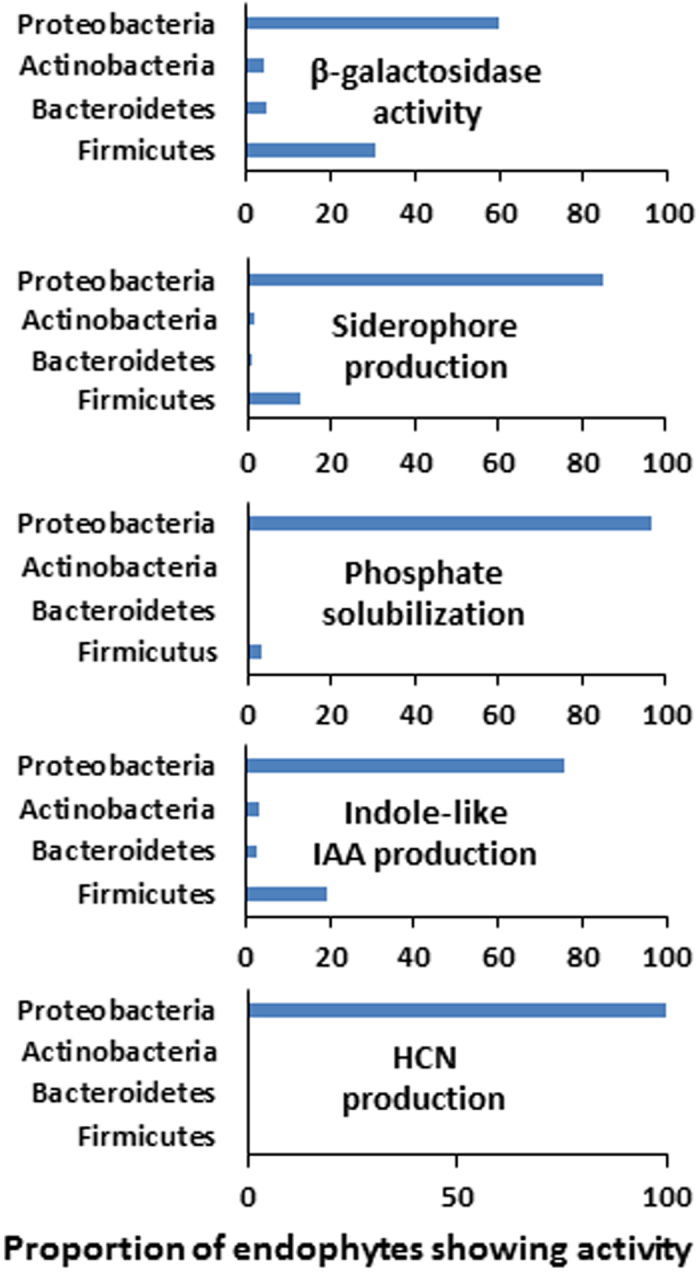
Graphs summarizing at the phylum level proportion of bacterial endophytes that showed potential plant-benefiting activities.

All the 252 bacterial endophytes isolated from MCGs were evaluated for their potential properties like plant growth promotion (siderophore production, phosphate solubilization, indole-3-acetic acid (IAA)-like indole derivatives production, hydrogen cyanide production and ß-glucosidase activity) (Supplementary Table S3). Among 252 isolates, 185 (73.41%), 118 (46.82%), 168 (66.66%), 32 (12.69%) and 98 (38.88%) bacterial isolates showed the positive result for siderophore production, phosphate solubilization, IAA-like indole derivatives production, hydrogen cyanide (HCN) production and ß-glucosidase activity, respectively. Among the isolates showing each activity, *Proteobacteria* accounted for 84.86, 96.61, 75.60, 100 and 60.20% of isolates possessing siderophore production, phosphate solubilization, IAA-like indole derivatives production, HCN production and ß-glucosidase activity, respectively (Fig. 5). *Firmicutes* were also enriched in isolates showing ß-glucosidase activity, siderophore production, and IAA production. Most of isolates belonging to *Actinobacteria* and *Bacteroidetes*, however, did not display such activities in our assay. Notably, HCN production was detected only in *Proteobacteria*. Except HCN production, location C, which showed the highest species richness and diversity, was the site where number of isolates having different activities were most abundant (Supplementary Fig. S6). Number of HCN-producing isolates was the highest in the location U.

## Discussion

Despite importance of ginseng as a medicinal plant, little is known about its endophytic community. Considering emerging roles of endopytes in plant development, growth, fitness, and diversification, such knowledge gap needs to be filled for exploitation of endophytes in better production of ginseng and its important metabolites. To this end, one objective of this study was to examine the community composition of bacterial endophytes from MCG collected from 24 different sites. To the best of our knowledge, our study is the first report on isolation and identification of bacterial endophytes in MCGs. Here we took culture-dependent approach, since our final goal is to build working collection of endophytes that can be explored for their potentially beneficial properties toward ginseng plants. Although limitation of culture-dependent approach in surveying composition of microbiome is well-known, it has been realized that a good number of bacterial endophytes can be isolated and cultured for characterization and that microbes in endosphere and rhizosphere are more amenable to culture than microbes in bulk soil^17^. Thus, it is likely that our data provide insights into endobiome composition and diversity in ginseng plants. However, it should be noted that our endophyte collection might not be a complete reflection of what plants have inside them.

In this work, we obtained 1,886 different isolates from MCGs, which were grouped into 252 isolates based on 16S rDNA sequences. Taxonomic binning revealed that they comprise 117 OTUs belonging to 35 different genera including *Pseudomonas*, *Bacillus* and *Paenibacillus*. Among these, 14 genera (*Pseudomonas*, *Bacillus*, *Paenibacillus*, *Lysinibacillus*, *Micrococcus*, *Microbacterium*, *Arthrobacter*, *Serratia*, *Staphylococcus*, *Pantoea*, *Chryseobacterium*, *Erwinia*, *Stenotrophomonas* and *Sphingobium*) were reported previously as endophytes in field-grown ginseng^23,24^. Nineteen genera including *Psychrobacillus*, *Burkholderia*, *Sphingomonas*, *Methylobacterium*, *Acinetobacter* and *Raoultella*, were not reported previously as endophytes in field-ginseng but reported as endophytes in other plant species^25,26^. These data may suggest that MCGs harbor more diverse array of endophytes than field-grown ginseng, which is subjected to uniform cultivation practice and intensive use of agrochemicals.

Microbial communities associated with plants are shaped by a wide variety of host and environmental factors, including plant genotypes, geographic locations, and edaphic factors^3^. Here we observed substantial variations in bacterial endophyte communities among sampling sites, leading to any two sites sharing very small proportion of bacterial OTUs. Our analyses and comparisons across sampling sites suggested that geography, climates, and soil chemistry might not be a major driving force in shaping bacterial endobiome communities in MCGs. This result suggests that endemism in combination with stochastic nature of endosphere colonization may be a community-shaping force operating for bacterial endophytes in MCG. It is likely that dispersal limitation resulted in such endemism. The fact that MCG-growing sites are located at remote places where there is little disturbance lends further support to this hypothesis. However, it should be noted that measurements of edaphic and climatic factors might not accurately represent environmental conditions of individual sampling sites. It should be also noted that genotype data are not available, although it is generally assumed that little genetic variations exist among MCGs.

Association network analysis can reveal potential ecological relationships such as symbiosis and antagonism among microbes associated with plants^22^. Our analysis showed that most of relationships are mutual exclusion. However, it seems that many of mutual exclusion relationships found in our analysis are probably not reflection of true ecological relationships among microbes but rather reflection of markedly different endophyte composition in sampling sites. Despite such limitation, some of the relationships uncovered in our analysis are worth noting. For example, *R*. *ornithinolytica* and *B*. *stabilis* individually showed co-occurrence relationship with *P*. *tremae*. Although we did not know whether co-occurrence of each pair of bacterial endophytes has any ecological implication for now, it is tempting to further test their relationship and its impact on ginseng plants.

It is known that different tissues, due to the differences in the anatomical structure and physiological conditions, harbor different types of endophytes^26^. When we investigated bacterial endophytes from leaf, stem and root of MCG, we found that some isolates were tissue specific, while others were recovered from all the tissues. There is evidence that root is the potential entry site for bacterial endophytes that can migrate to the above-ground parts during plant development^27,28^. In line with this, there were 41 OTUs that were present in all tissues, suggesting that at least some, if not all, might have derived from endophytes that invaded root tissue. However, we also found significantly more number of leaf-specific endophytes, suggesting that there are other important routes into ginseng endosphere. One of possible portals is ginseng seed. Future research is thus required to evaluate the microbiome associated with the seeds and cross-generational propagation of endophytic microbes.

Plant-associated microbes may be harmful or beneficial to the plants. The ability of bacterial endophytes having growth-promoting properties and bio-transforming activities of compounds has been of great interest to those who aim to exploit endophytes for new paradigm of agriculture. Plant growth promoting bacteria (PGPB) regulate the growth promotion directly or indirectly by fixing biological nitrogen, producing siderophore, solubilizing phosphorus, and IAA-like indole derivatives^29^. Here we examined all the isolates for their potential growth promoting properties as well as the activity of HCN production and ß-glucosidase. Siderophore production by endophytes may be a common phenomenon for sequestering irons, which is beneficial to plants due to the inhibition of plant pathogen growth and increased availability of iron for plants themselves^30,31^. In this study, siderophore production was detected in more than half of the isolates (73.41%), among which majority of them were *Proteobacteria* (84.86%). Phosphate is a major essential macronutrient for plant growth and development and applied to the soil as inorganic fertilizer, which is unavailable to the plants. Several reports indicated that bacterial endophytes have ability to solubilize inorganic phosphate so that plant can easily uptake the soluble phosphate^32^. We found that 46.82% of the isolates showed the phosphate solubilization activity. Absolute majority of them belongs to *Proteobacteria* (96.61%). IAA, a phytohormone auxin, is essential for plant growth and development. Some bacterial endophytes were known to have ability to synthesize IAA-like indole derivatives, which are responsible for plant growth promotion^33^. In this study we detected from more than half of bacterial endophytes (75.60%), the ability to produce IAA-like indole derivatives. It is interesting that IAA is a plant hormone and has no apparent function in bacterial cells. It was speculated that IAA could improve the fitness of the plant microbe interaction. Many plant-associated and rhizobacteria have capability to produce HCN, a volatile secondary metabolite, which suppresses the development of microorganisms^34^. It also affects the growth and development of plants negatively. We found that small number of our bacterial endophytes (12.69%) all belonging to the genus *Pseudomonas* showed HCN production. Previous reports reveled that HCN production is a common trait of *Pseudomonas* spp.^35^ HCN is a good suppressor of nematode and some fungi^36^, although it is also harmful for plant. The activity of ß-glucosidase in microorganisms can convert major ginsenosides to minor form of ginsenosides, as they are the most pharmaceutically active compounds^37^. Among 252 isolates, we found 98 (38.88%) isolates showed positive result for ß-glucosidase activity.

Taken together, our extensive analyses on the culturable bacterial endophytes in MCG suggested that MCG endosphere harbors diverse bacterial species that are not influenced by biogeography of the host species. We showed that endophyte composition is markedly different from one site to another and also from one tissue to another. Knowledge on the composition and diversity of bacterial endophytes as well as working collection of them will provide insights and experimental basis for future applications of these endophytes to plant health promotion, phytoremediation and bio-transformation. In addition, it will be interesting to investigate dynamics of and interaction among endophytes in MCGs. These efforts will provide empirical baseline to further deepen our understanding of the complex plant-microbe interactions and to facilitate precise and target-oriented application.

## Materials and Methods

### Site description and sampling

Four-year-old MCGs (*Panax ginseng* Meyer) were harvested from undisturbed mountain areas of 24 different sites of 6 provinces in Republic of Korea during the growing season, June to August, 2013 (Supplementary Fig. S1). Disease-free healthy looking 10 plants were collected from each site. Following sampling, the plants were immediately brought to the laboratory using a wooden box with some wet moss to keep them fresh. The samples were preserved at 4 °C and endophytes were isolated from all the samples within 24 h of collection.

### Sample preparation

Bacterial endophytes were isolated from root, stem, and leaf tissues of ginseng plants. The samples were surface sterilized using the following protocol with modification^23^. The ginseng plants were washed well under running tap water to remove surface soil and other inert particles. Ginseng plant was separated into three tissues (root, stem, leaf) and cut into a small pieces (root and stem, 1.0 cm; leaf, 0.5 cm). Surface sterilization of the tissue samples was done stepwise by immersing in 70% ethanol for 30 s, 2% sodium hypochlorite (NaOCl) for 10 min, and 70% ethanol for 30 s, followed by three rinses in sterile distilled water to remove the surface sterilization agent. After surface sterilization, the samples were kept between 2 layers of the sterilized tissue papers for proper drying. All the procedures were done under aseptic condition in laminar air flow bench. After drying, six to seven tissue segments were placed separately on to tryptic soy agar (TSA) plate.

Two successful disinfection processes were done to verify the efficacy of sterilization procedure. First, the aliquot (100 µL) of the water from the last rinse was spread on TSA plate and second, the sterilized tissue samples were pressed onto TSA plate and transfer to the new TSA plate for endophyte isolation. The plates were examined for bacterial growth after incubation at 28 °C for 2–3 d.

### Isolation and molecular identification of bacterial endophytes

All the plates were incubated at 28 °C and observed up to 15 d. Individual bacterial colonies from the tissue samples were transferred to new TSA plate and purified by streak plate method. Several sub-cultures were done by streak plate method to confirm and maintain pure culture. After getting pure culture, bacterial isolates were grouped by carefully examining on-plate phenotypes: colony color, form, size, opaqueness, texture, surface, edge, and height.

A total 1,886 isolates were selected based on morphological characteristics. Two sets of universal primers (27 F/1492 R and 337 F/1392 R) were used to amplify the bacterial 16S rDNA^38^. The sequences of 16 S rDNA were amplified from single colonies according to the protocol of the company COSMO Genetech (Seoul, Korea). All 16 S rDNA sequences generated in this study (Supplementary Dataset 1) were analyzed by EZ taxon (http://eztaxon-e.ezbiocloud.net/)^39^ and BLAST search with the sequences available in the GenBank database (http://blast.ncbi.nlm.nih.gov/) to assign OUT. Phylogenetic tree was constructed using neighbor-joining method in MEGA6 (http://www.megasoftware.net). The bootstrap analysis was performed with 1,000 replications to assess the relative stability of the branches.

### Determination of species abundance and evenness

Relative abundance was calculated as a percentage of an isolate belonging to a particular isolate group, species or phylum compared to all isolates recovered from all the tissue samples. Species richness (S) is the number of species recovered from the particular site or tissue. Species evenness (E) was calculated as E = H/H_*max*_ · H_*max*_ is the maximum possible value of Shannon’s diversity index, *H*. *H* = − Σ (P*i* × In P*i*) while P*i* is the proportional abundance of species *i* in particular tissue or site^40^.

### Analyses of statistics and association

All the statistical analysis of data were performed using R programming (https://www.R-project.org/). Microbial association network was constructed using the CoNet available as an app within Cytoscape^41^.

### Examination of potential properties of bacterial endophytes

#### Siderophor production

Bacterial isolates were assayed for the production of siderophore on universal chrome azurol S (CAS) agar plate. The isolates were spot inoculated in the CAS agar plate and incubated at 28 °C for 3–5 d. Yellow to orange halo development around the colony was considered as a positive result.

#### Phosphate solubilization

All the isolates were checked for the ability to solubilize the insoluble phosphate using the following protocol. Bacterial isolates were spot inoculated on Pikovskaya’s agar plates and incubated at 28 °C for 4 d. Clear zone around the bacterial colony was considered as a positive result for solubilization of insoluble phosphate.

#### IAA-like indole derivative production

All the bacterial isolates were screened for their ability to produce phytohormone IAA-like indole derivatives according to the following method with modification^42^. Briefly, single colony of bacterial isolate was inoculated in 10 mL of tryptic soy broth (TSB) supplemented with 2 mg mL^−1^ of tryptophan and incubated at 28 °C for 72 h at 150 rpm. Indole derivatives were measured by mixing 1 mL of broth with 1 mL of Salkowsky’s reagent. Development of pink to violet color was considered as a positive color for IAA-like indole derivative production.

### HCN production

All the isolates were screened for the production of HCN according to the following method with modification^43^. Briefly the TSB medium was amended with 4.4 g glycine L^−1^ and the isolates were spot inoculated on the medium. A piece of Whatman filter paper (GE Whatman, Pittsburgh, PA, USA) previously soaked in 2% sodium carbonate in 0.5% picric acid solution was placed on the upper lid of the plate and incubate at 28 °C for 4 d. HCN production was indicated with the change of the color from yellow to orange to red.

### β-glucosidase activity

All the isolates were checked for β-glucosidase activity. The isolates were spot inoculated on the esculin iron agar (EIA) plate (Sigma-Aldrich, St Louis, MO, USA)^44^ and incubated at 28 °C for 48 h. Brown to dark brown colors around the bacterial colony were considered as a positive result of the enzyme activity.

## Electronic supplementary material

Supplementary Figures and Tables

Supplementary Dataset 1
