# Publisher Correction: Composition, diversity and bioactivity of culturable bacterial endophytes in mountain-cultivated ginseng in Korea

**DOI:** 10.1038/s41598-018-22623-z

**Published:** 2018-03-06

**Authors:** MD. Emran Khan Chowdhury, Junhyun Jeon, Soon Ok Rim, Young-Hwan Park, Seung Kyu Lee, Hanhong Bae

**Affiliations:** 1 Department of Biotechnology, Yeungnam University, Gyeongsan, Gyeongbook 38541 Republic of Korea; 2 Division of Forest Diseases & Insect Pests, Korea Forest Research Institute, Seoul, 02455 Republic of Korea

Correction to: *Scientific Reports* 10.1038/s41598-017-10280-7, published online 30 August 2017

The original version of this Article contained an error in the spelling of the author MD. Emran Khan Chowdhury, which was incorrectly given as Emran Khan Chowdhury. This has now been corrected in the PDF and HTML versions of the Article.

