# Supplementary material for: Composition, diversity and bioactivity of culturable bacterial endophytes in mountain-cultivated ginseng in Korea
Source: Sci Rep. 2017 Aug 30;7:10098. doi: 10.1038/s41598-017-10280-7 (PMC5577135; doi:10.1038/s41598-017-10280-7)
Supplement: Supplementary file 1 — Supplementary Figures and Tables [file 41598_2017_10280_MOESM1_ESM.pdf]

Composition, diversity and bioactivity of culturable bacterial endophytes in mountain-cultivated ginseng in Korea

Md. Emran Khan Chowdhury[1¶], Junhyun Jeon[1¶], Soon Ok Rim[1¶], Young-Hwan Park[1], Seung Kyu Lee[2], Hanhong Bae[1,*]

[1] Department of Biotechnology, Yeungnam University, Gyeongsan, Gyeongbook 38541, Republic of Korea

[2] Division of Forest Diseases & Insect Pests, Korea Forest Research Institute, Seoul 02455, Republic of Korea

[¶]These authors contributed equally to the work.

[*] Correspondence:

Hanhong Bae

Professor, Department of Biotechnology, Yeungnam University, Gyeongsan 38541, Republic of Korea, Phone: 8253-810-3031 (office), Fax: 8253-810-4769, Email: hanhongbae@ynu.ac.kr

Keywords: bacterial endophyte, mountain-cultivated ginseng, diversity, composition

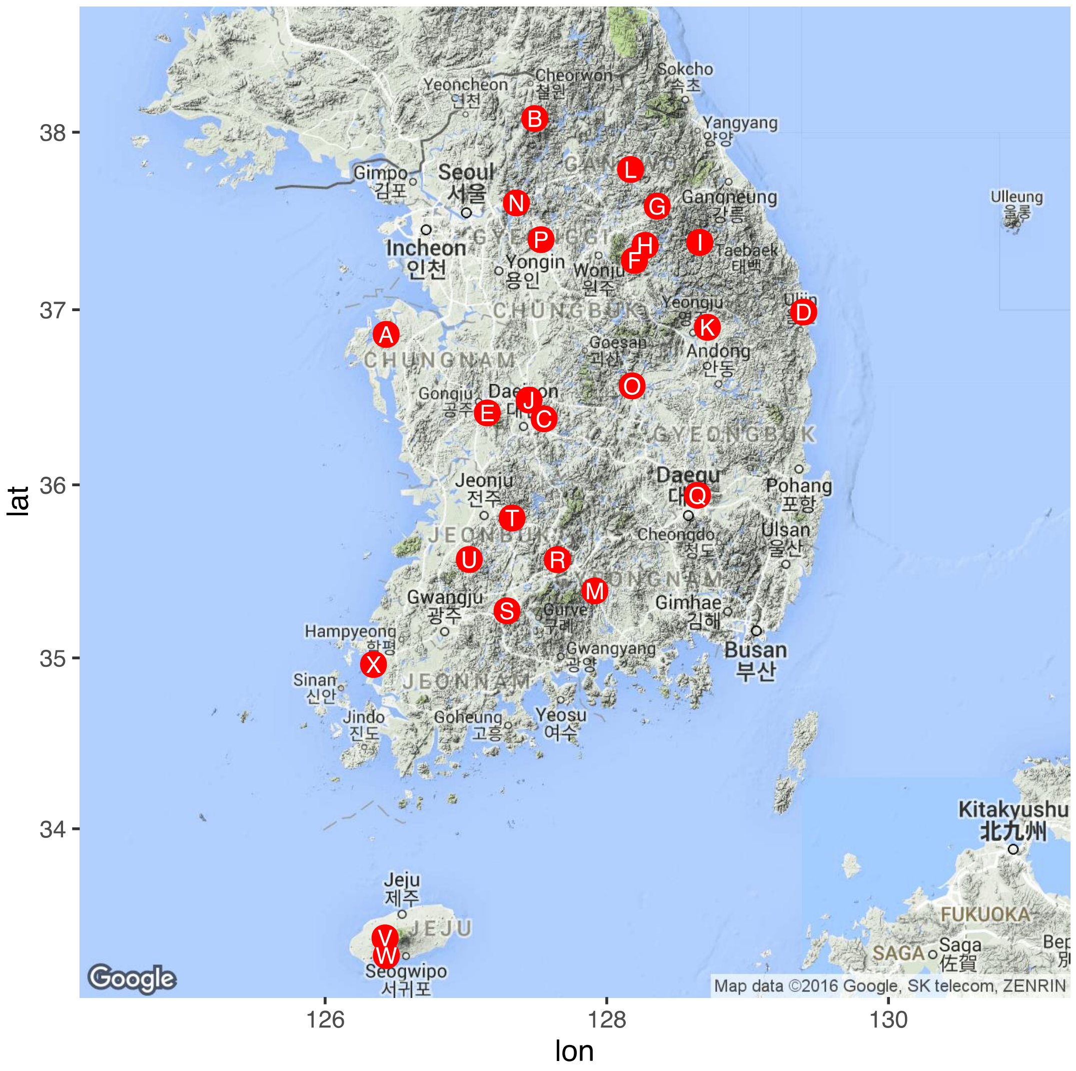

Supplementary Fig 1. A map showing locations of 24 sampling sites in South Korea. Each circle represents sampling site labeled as A to X. Google map was modified to indicate sampling locations using ggmap package available in R programming (http://www.R-project.org).

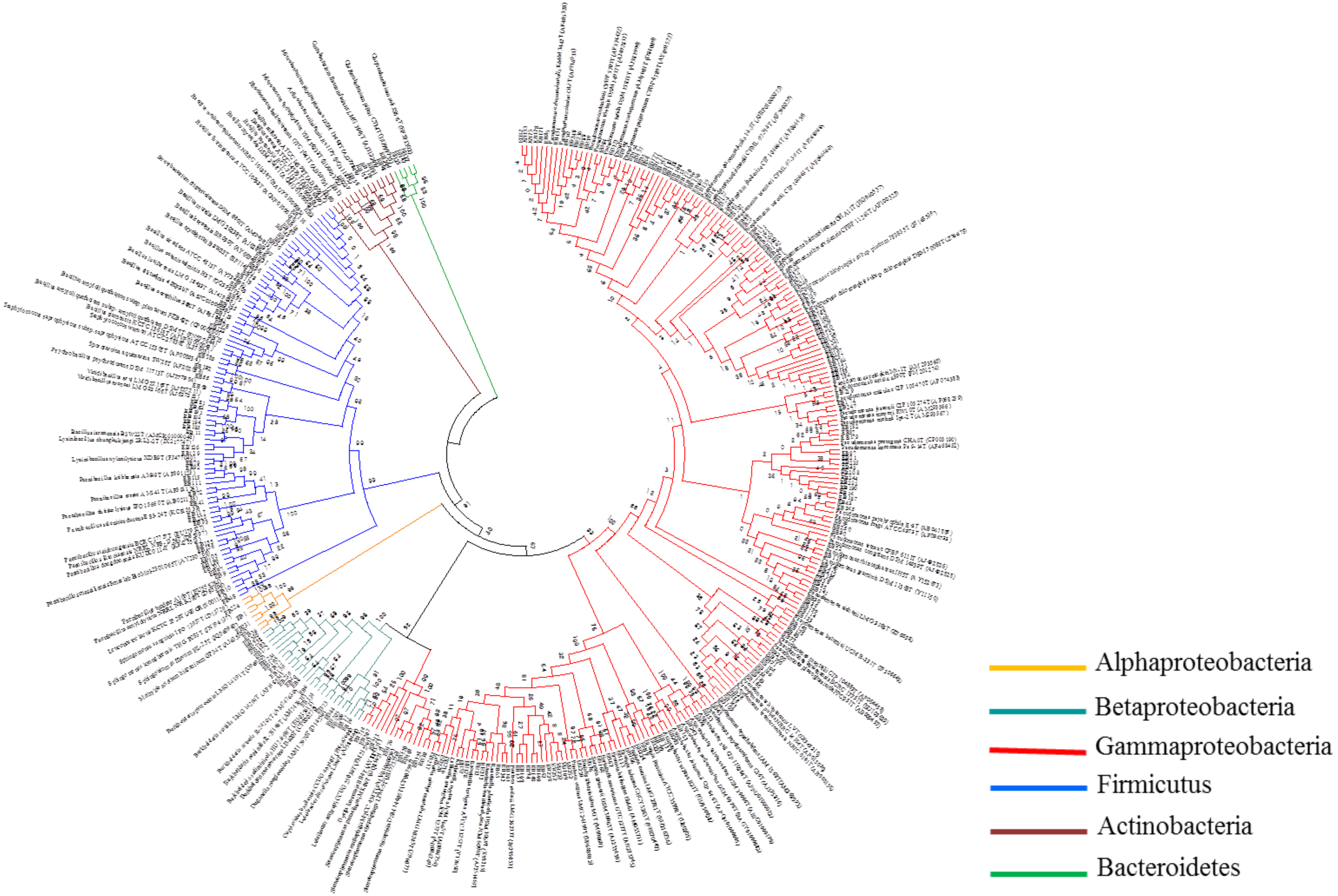

Supplementary Figure S2. Phylogenetic tree showing the placement of different isolates with reference strains based on 16S rDNA sequence. The phylogenetic tree was generated by neighbor-joining methods using ClustlW and Mega6. Tamura-Nei method was used to measure the evolutionary distance. Bootstrap values expressed as percentage of 1,000 replications are indicated at the nodes.

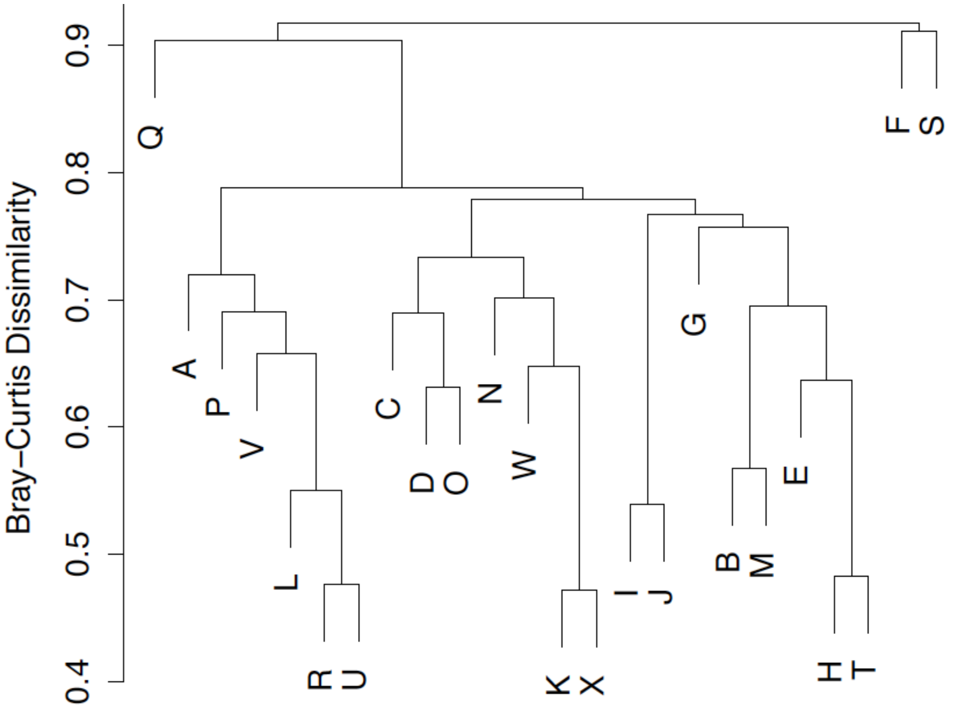

Supplementary Figure S3. Clustering of sampling sites based on Bray-Curtis Dissimilarity. The dissimilarity measure was calculated using composition data of bacterial endophytes in each site.

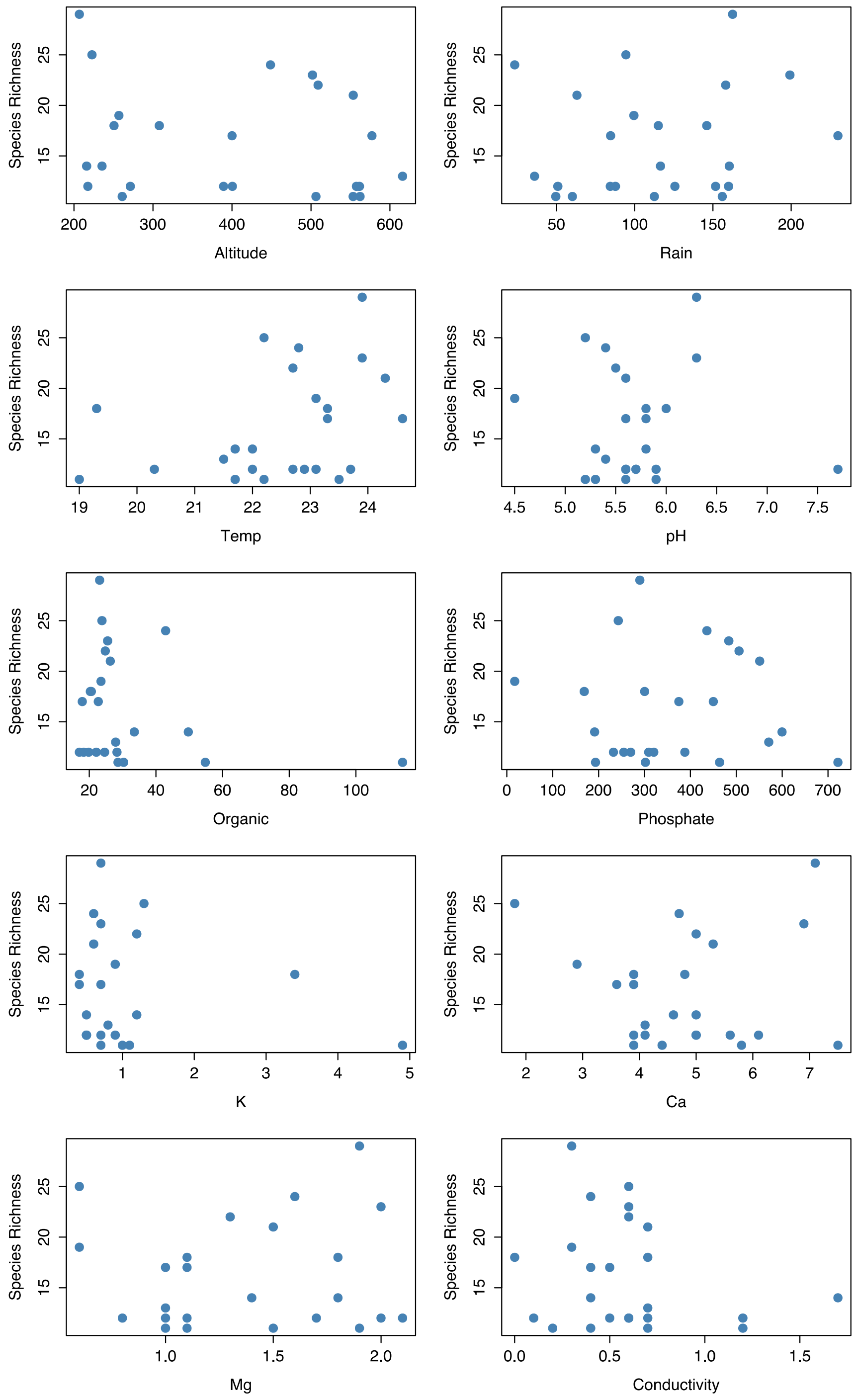

Supplementary Figure S4. Scatter plots showing relationships between meta-data of sampling sites and species richness.

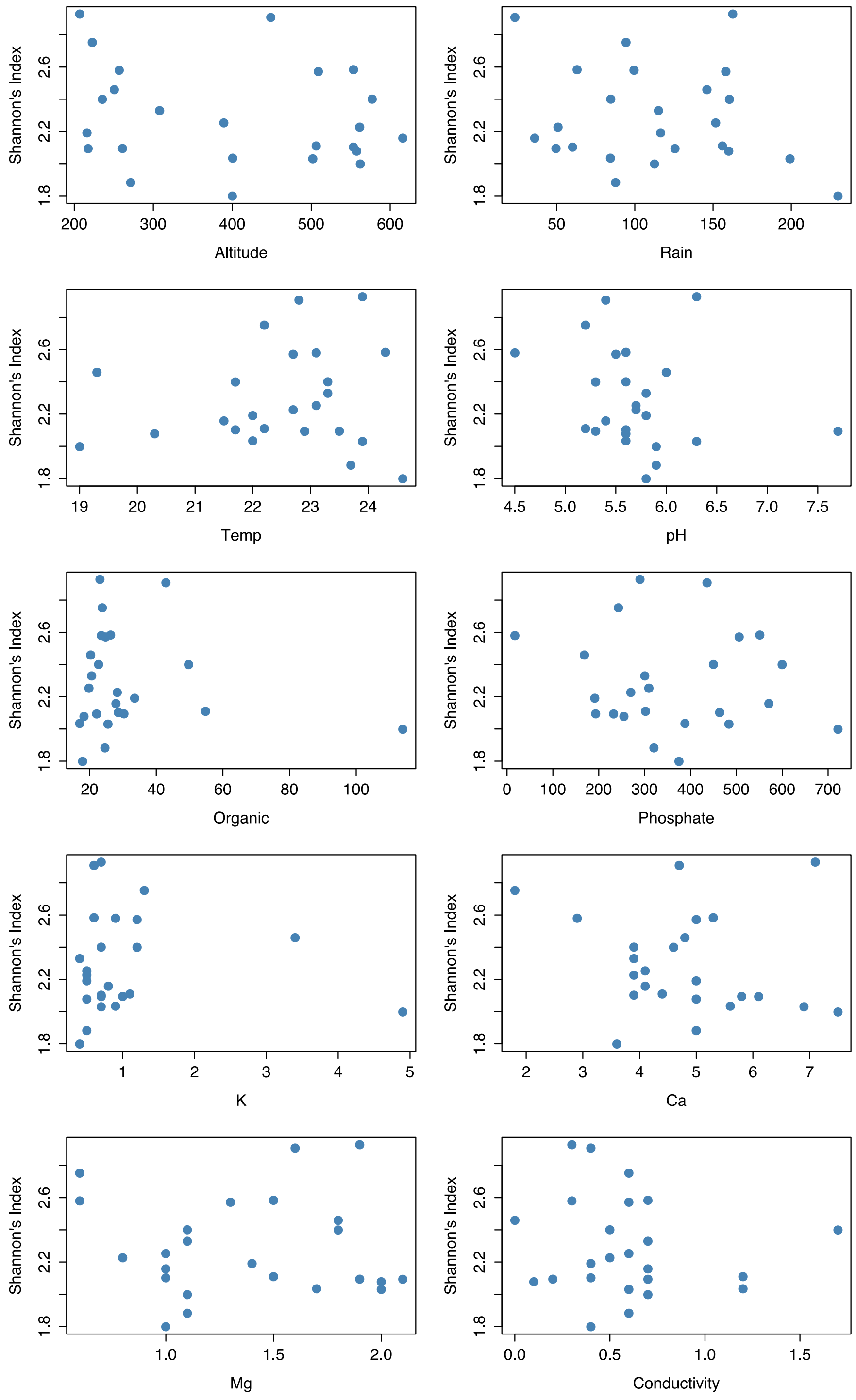

Supplementary Figure S5. Scatter plots showing relationships between meta-data of sampling sites and Shannon's index.

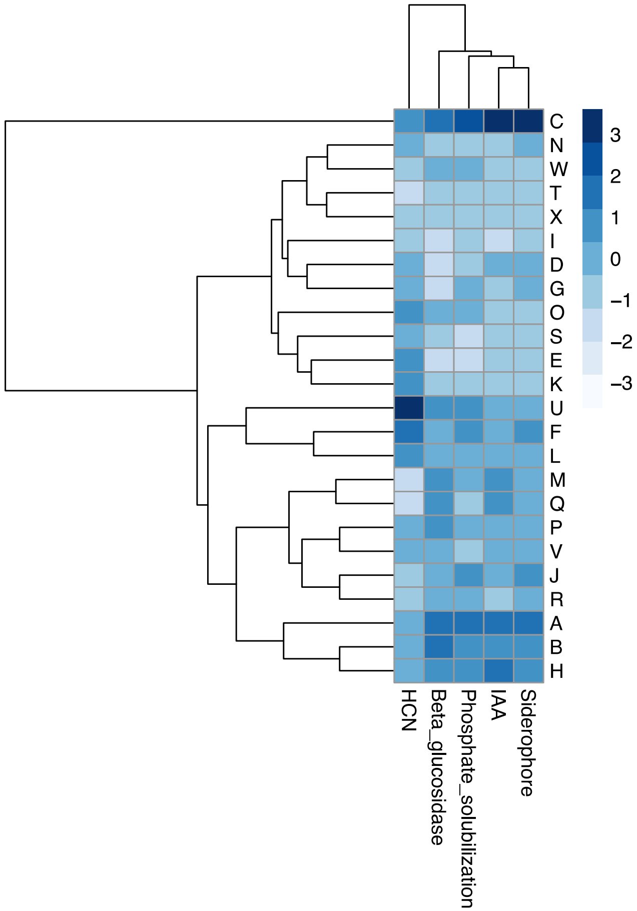

Supplementary Figure S6. A heat-map showing relationships between locations (rows: A to X) and biological activities (column) of endophytic bacteria isolated from ginseng plants. Scale (color-coded) indicates normalized (column) number of isolates having designated activity. Clustering was carried out using 'average' method based on euclidean distance.

Supplementary Table S1. Closest relatives of bacterial endophytes isolated from mountain-cultivated ginseng plants based on the 16S rDNA sequence analyzed by EzTaxon.

Isolate	Reference strain with accession number	Similarity (%)
EB1	*Sphingomonas kyungheensis* (JN196137)	99.92
EB2	*Bacillus toyonensis* (CP006863)	99.93
EB3	*Pseudomonas lurida* (AJ581999)	99.71
EB4	*Pseudomonas lurida* (AJ581999)	99.70
EB5	*Bacillus mycoides* (ACMU01000002)	99.93
EB6	*Burkholderia arboris* (AM747630)	99.93
EB7	*Bacillus toyonensis* (CP006863)	99.85
EB8	*Burkholderia arboris* (AM747630)	99.93
EB9	*Paenibacillus amylolyticus* (D85396)	99.57
EB10	*Paenibacillus amylolyticus* (D85396)	99.64
EB11	*Bacillus isronensis* (AMCK01000046)	99.29
EB12	*Raoultella terrigena* (Y17658)	99.86
EB13	*Micrococcus endophyticus* (EU005372)	99.93
EB14	*Pseudomonas koreensis* (AF468452)	99.92
EB15	*Bacillus siamensis* (AJVF01000043)	100.0
EB16	*Cupriavidus basilensis* (FN597608)	99.71
EB17	*Cupriavidus basilensis* (FN597608)	99.71
EB18	*Pseudomonas grimontii* (AF268029)	99.93
EB19	*Bacillus toyonensis* (CP006863)	99.93
EB20	*Pseudomonas graminis* (Y11150)	98.79
EB21	*Pseudomonas koreensis* (AF468452)	100.0
EB22	*Burkholderia pyrrocinia* LMG 14191(U96930)	97.70
EB23	*Pseudomonas extremaustralis* (AHIP01000073)	99.85
EB24	*Pseudomonas lurida* (AJ581999)	99.78
EB25	*Chryseobacterium piperi* (EU999735)	98.28
EB26	*Pseudomonas graminis* (Y11150)	99.05
EB27	*Chryseobacterium soli* (EF591302)	99.78

EB28	*Pseudomonas putida* (AP013070)	99.86
EB29	*Pseudomonas baetica* (FM201274)	99.64
EB30	*Stenotrophomonas rhizophila* (CP007597)	99.93
EB31	*Bacillus muralis* (AJ628748)	100.0
EB32	*Pseudomonas lurida* (AJ581999)	99.78
EB33	*Pseudomonas lurida* (AJ581999)	99.60
EB34	*Pseudomonas rhodesiae* (AF064459)	99.41
EB35	*Pseudomonas putida* (AP013070)	99.14
EB36	*Methylobacterium hispanicum* (AJ635304)	99.92
EB37	*Pseudomonas protegens* (CP003190)	100.0
EB38	*Pseudomonas moorei* (AM293566)	98.45
EB39	*Pseudomonas azotoformans* (D84009)	99.77
EB40	*Burkholderia stabilis* (AF148554)	99.93
EB41	*Paenibacillus chitinolyticus* (AB021183)	99.78
EB42	*Pantoea rodasii* (JF295053)	98.81
EB43	*Pseudomonas fragi* (AF094733)	99.64
EB44	*Duganella zoogloeoides* (D14256)	99.42
EB45	*Pseudomonas psychrotolerans* (AJ575816)	100.0
EB46	*Pseudomonas koreensis* (AF468452)	99.57
EB47	*Chryseobacterium piperi* (EU999735)	98.45
EB48	*Leuconostoc lactis* (AEOR01001150)	100.0
EB49	*Pseudomonas koreensis* (AF468452)	100.0
EB50	*Pseudomonas simiae* (AJ936933)	100.0
EB51	*Pseudomonas helmanticensis* (HG940537)	99.75
EB52	*Pseudomonas congelans* (AJ492828)	99.93
EB53	*Acinetobacter soli* (APPU01000012)	99.93
EB54	*Pseudomonas hunanensis* (JX545210)	99.78
EB55	*Sporosarcina aquimarina* (AF202056)	99.07
EB56	*Microbacterium phyllosphaerae* (AJ277840)	99.77
EB57	*Klebsiella oxytoca* (AB004754)	99.21
EB58	*Pseudomonas putida* (AP013070)	99.60

EB59	*Erwinia persicina* (U80205)	99.50
EB60	*Rhodococcus baikonurensis* (AB071951)	99.77
EB61	*Pseudomonas extremaustralis* (AHIP01000073)	99.85
EB62	*Viridibacillus arenosi* (AJ627212)	99.86
EB63	*Pseudomonas grimontii* (AF268029)	99.71
EB64	*Pseudomonas psychrophila* (AB041885)	99.64
EB65	*Raoultella ornithinolytica* (AJ251467)	99.71
EB66	*Pseudomonas reinekei* (AM293565)	99.49
EB67	*Ewingella americana* (AB273745)	98.44
EB68	*Curtobacterium flaccumfaciens* (AJ312209)	100.0
EB69	*Psychrobacillus psychrodurans* (AJ277984)	97.87
EB70	*Bacillus toyonensis* (CP006863)	100.0
EB71	*Pseudomonas monteilii* (AF064458)	99.79
EB72	*Paenibacillus terrae* (AF391124)	99.93
EB73	*Burkiholderia sediminicola* (EU035613)	99.04
EB74	*Acinetobacter calcoaceticus* (AIEC01000170)	100.0
EB75	*Pseudomonas lurida* (AJ581999)	99.71
EB76	*Sodalis glossinidius* (M99060)	96.50
EB77	*Pseudomonas plecoglossicida* (AB009457)	98.69
EB78	*Bacillus aryabhattai* (EF114313)	99.44
EB79	*Lysinibacillus xylanilyticus* (FJ477040)	100.0
EB80	*Bacillus anthracis* (AB190217)	99.77
EB81	*Viridibacillus arvi* (AJ627211)	100.0
EB82	*Pseudomonas chlororaphis* (FJ168539)	99.11
EB83	*Pseudomonas plecoglossicida* (AB009457)	98.78
EB84	*Pseudomonas simiae* (AJ936933)	99.73
EB85	*Pseudomonas lurida* (AJ581999)	99.93
EB86	*Pseudomonas extremaustralis* (AHIP01000073)	99.84
EB87	*Pseudomonas koreensis* (AF468452)	99.93
EB88	*Pseudomonas azotoformans* (D84009)	99.85
EB89	*Pseudomonas grimontii* (AF268029)	99.85

EB90	*Pseudomonas tremae* (AJ492826)	99.55
EB91	*Curtobacterium flaccumfaciens* LMG 3645 (AJ312209)	99.63
EB92	*Paenibacillus kribbensis* (AF391123)	99.65
EB93	*Paenibacillus taichungensis* (EU179327)	99.65
EB94	*Pantoea rodasii* (JF295053)	98.81
EB95	*Bacillus oceanisediminis* (GQ292772)	99.64
EB96	*Bacillus koreensis* (AY667496)	100.0
EB97	*Paenibacillus tundrae* (EU558284)	99.29
EB98	*Bacillus aerophilus* (AJ831844)	100.0
EB99	*Erwinia persicina* (U80205)	99.63
EB100	*Pseudomonas cremoricolorata* (AB060136)	100.0
EB101	*Stenotrophomonas rhizophila* (CP007597)	99.52
EB102	*Bacillus circulans* (AY724690)	99.07
EB103	*Viridibacillus arenosi* (AJ627212)	100.0
EB104	*Pseudomonas putida* (AP013070)	99.86
EB105	*Paenibacillus massiliensis* (AY230766)	99.42
EB106	*Erwinia toletana* (FR870447)	98.28
EB107	*Pseudomonas taiwanensis* (EU103629)	99.69
EB108	*Pseudomonas lurida* (AJ581999)	99.64
EB109	*Pantoea rodasii* (JF295053)	98.81
EB110	*Curtobacterium flaccumfaciens* (AJ312209)	100.0
EB111	*Paenibacillu skribbensis* (AF391123)	99.64
EB112	*Bacillus altitudinis* (ASJC01000029)	100.0
EB113	*Erwinia billingiae* (AM055711)	99.86
EB114	*Pseudomonas moorei* (AM293566)	98.88
EB115	*Paenibacillus kribbensis* (AF391123)	99.69
EB116	*Bacillus weihenstephanensis* (BAUY01000093)	100.0
EB117	*Paenibacillus selenitireducens* (KC815539)	99.22
EB118	*Pseudomonas koreensis* (AF468452)	98.77
EB119	*Bacillus aryabhattai* (EF114313)	100.0
EB120	*Stenotrophomonas maltophilia* (JALV01000036)	98.68

EB121	*Pseudomonas lurida* (AJ581999)	99.64
EB122	*Bacillus amyloliquefaciens* (CP000560)	99.93
EB123	*Pseudomonas veronii* (AF064460)	99.79
EB124	*Stenotrophomonas panacihumi* (GQ856217)	98.62
EB125	*Bacillus luciferensis LMG 18422* (AJ419629)	99.70
EB126	*Lysinibacillus chungkukjangi* (JX217747)	99.93
EB127	*Pseudomonas grimontii* (AF268029)	99.93
EB128	*Lelliottia amnigena* (AB004749)	99.93
EB129	*Lysinibacillus xylanilyticus* (FJ477040)	99.93
EB130	*Pseudomonas koreensis* (AF468452)	99.59
EB131	*Pseudomonas extremorientalis* (AF405328)	99.80
EB132	*Pseudomonas batumici* (JF306642)	99.43
EB133	*Pseudomonas trivialis* (AJ492831)	99.64
EB134	*Burkholderia sediminicola* (EU035613)	99.85
EB135	*Pseudomonas lurida* (AJ581999)	99.78
EB136	*Erwinia persicina* (U80205)	99.43
EB137	*Pseudomonas lurida* (AJ581999)	99.47
EB138	*Pseudomonas lurida* (AJ581999)	99.53
EB139	*Pseudomonas chlororaphis* (FJ168539)	98.79
EB140	*Pantoea rodasii* (JF295053)	98.88
EB141	*Pseudomonas cichorii* (Z76658)	98.81
EB142	*Pseudomonas batumici* (JF306642)	98.99
EB143	*Pseudomonas putida* (AP013070)	99.85
EB144	*Pseudomonas migulae* (AF074383)	99.62
EB145	*Bacillus cereus* (AE016877)	100.0
EB146	*Pseudomonas chlororaphis* (FJ168539)	99.11
EB147	*Pseudomonas graminis* (Y11150)	99.33
EB148	*Pantoea rodasii* (JF295053)	98.81
EB149	*Pseudomonas graminis* (Y11150)	99.93
EB150	*Pseudomonas grimontii* (AF268029)	99.64
EB151	*Pseudomonas koreensis* (AF468452)	99.64

EB152	*Pseudomonas koreensis* (AF468452)	99.64
EB153	*Acinetobacter oryzae* (GU954428)	99.43
EB154	*Pseudomonas lurida* (AJ581999)	99.71
EB155	*Paenibacillus illinoisensis* (D85397)	97.31
EB156	*Paenibacillus dongdonensis (KF425513)*	98.26
EB157	*Pantoea rodasii* (JF295053)	98.88
EB158	*Curtobacterium flaccumfaciens* (AJ312209)	99.56
EB159	*Burkholderia stabilis* (AF148554)	99.71
EB160	*Pseudomonas taiwanensis* (EU103629)	99.78
EB161	*Raoultella ornithinolytica* (AJ251467)	99.86
EB162	*Pseudomonas jessenii* (AF068259)	99.42
EB163	*Pseudomonas brenneri* (AF268968)	99.56
EB164	*Pseudomonas koreensis* (AF468452)	99.57
EB165	*Pseudomonas koreensis* (AF468452)	99.71
EB166	*Pseudomonas baetica* (FM201274)	99.56
EB167	*Pseudomonas koreensis* (AF468452)	99.64
EB168	*Serratia grimesii* (AJ233430)	99.78
EB169	*Pantoea rodasii* (JF295053)	98.81
EB170	*Pseudomonas lurida* (AJ581999)	99.64
EB171	*Pseudomonas extremorientalis* (AF405328)	99.93
EB172	*Burkholderiastabilis* (AF148554)	99.93
EB173	*Pseudomonas lurida* (AJ581999)	99.71
EB174	*Pseudomonas rhodesiae* (AF064459)	99.42
EB175	*Pseudomonas batumici* (JF306642)	99.12
EB176	*Stenotrophomonas maltophilia* (JALV01000036)	99.28
EB177	*Pseudomonas lurida* (AJ581999)	99.66
EB178	*Pseudomonas costantinii* (AF374472)	99.71
EB179	*Pseudomonas protegens* (CP003190)	100.0
EB180	*Pseudomonas baetica* (FM201274)	99.76
EB181	*Pseudomonas lurida* (AJ581999)	99.64
EB182	*Pseudomonas protegens* (CP003190)	100.0

EB183	*Pseudomonas putida* (AP013070)	99.84
EB184	*Pseudomonas koreensis* (AF468452)	99.60
EB185	*Bacillus isronensis* (AMCK01000046)	99.36
EB186	*Pseudomonas putida* (AP013070)	99.70
EB187	*Pseudomonas koreensis* (AF468452)	99.93
EB188	*Pseudomonas koreensis* (AF468452)	99.71
EB189	*Staphylococcus warneri* (L37603)	100.0
EB190	*Pseudomonas koreensis* (AF468452)	99.86
EB191	*Viridibacillus arenosi* (AJ627212)	100.0
EB192	*Staphylococcus saprophyticus* (AP008934)	100.0
EB193	*Burkholderia stabilis* (AF148554)	100.0
EB194	*Viridibacillus arvi* (AJ627211)	99.78
EB195	*Burkholderia stabilis* (AF148554)	100.0
EB196	*Paenibacillus amylolyticus* (D85396)	99.43
EB197	*Brevibacterium frigoritolerans* (AM747813)	99.57
EB198	*Pseudomonas palleroniana* (AY091527)	99.78
EB199	*Pseudomonas mohnii* (AM293567)	99.15
EB200	*Pseudomonas taiwanensis* (EU103629)	99.86
EB201	*Paenibacillus amylolyticus* (D85396)	99.28
EB202	*Pseudomonas lurida* (AJ581999)	99.71
EB203	*Acinetobacter johnsonii* (APON01000005)	99.60
EB204	*Curtobacterium flaccumfaciens* (AJ312209)	99.69
EB205	*Cupriavidus basilensis* (FN597608)	99.62
EB206	*Cupriavidus basilensis* (FN597608)	99.62
EB207	*Luteibacter anthropi* (FM212561)	98.79
EB208	*Pseudomonas koreensis* (AF468452)	100.0
EB209	*Pseudomonas brenneri* (AF268968)	99.56
EB210	*Pseudomonas baetica* (FM201274)	99.60
EB211	*Pseudomonas thivervalensis* (AF100323)	99.49
EB212	*Raoultella ornithinolytica* (AJ251467)	100.0
EB213	*Dyella koreensis* (AY884571)	99.06

EB214	*Luteibacter rhizovicinus* (AJ580498)	99.78
EB215	*Pseudomonas graminis* (Y11150)	99.06
EB216	*Lelliottia nimipressuralis* (Z96077)	99.20
EB217	*Lelliottia nimipressuralis* (Z96077)	99.34
EB218	*Pseudomonas chlororaphis* (Z76673)	98.70
EB219	*Pseudomonas baetica* (FM201274)	99.75
EB220	*Lelliottia nimipressuralis* (Z96077)	99.42
EB221	*Erwinia persicina* (U80205)	99.64
EB222	*Pantoea rodasii* (JF295053)	98.54
EB223	*Pseudomonas koreensis* (AF468452)	99.68
EB224	*Pantoea eucrina* (EU216736)	100.0
EB225	*Paenibacillus taichungensis* (EU179327)	99.64
EB226	*Sphingomonas sanguinis* (D13726)	99.70
EB227	*Arthrobacter arilaitensis* (FQ311875)	99.63
EB228	*Pantoea rodasii* (JF295053)	98.88
EB229	*Pseudomonas koreensis* (AF468452)	99.64
EB230	*Pantoea vagans* (EF688012)	99.85
EB231	*Sphingobium sufflavum* (JQ060960)	97.07
EB232	*Pantoea vagans* (EF688012)	99.55
EB233	*Pseudomonas koreensis* (AF468452)	100.0
EB234	*Burkholderia stabilis* (AF148554)	99.93
EB235	*Pseudomonas koreensis* (AF468452)	100.0
EB236	*Pseudomonas azotoformans* (D84009)	99.78
EB237	*Pseudomonas koreensis* (AF468452)	99.71
EB238	*Burkholderia stabilis* (AF148554)	99.71
EB239	*Burkholderia stabilis* (AF148554)	99.71
EB240	*Burkholderia stabilis* (AF148554)	99.93
EB241	*Pseudomonas koreensis* (AF468452)	99.64
EB242	*Raoultella planticola* (X93215)	99.78
EB243	*Acinetobacter radioresistens* (BAGY01000082)	99.92
EB244	*Pseudomonas koreensis* (AF468452)	100.0

EB245	*Pseudomonas psychrophila* (AB041885)	100.0
EB246	*Pseudomonas grimontii* (AF268029)	99.86
EB247	*Pseudomonas mohnii* (AM293567)	99.93
EB248	*Pseudomonas extremorientalis* (AF405328)	100.0
EB249	*Pseudomonas chlororaphis* (FJ168539)	98.87
EB250	*Pseudomonas tremae* (AJ492826)	99.49
EB251	*Pseudomonas lurida* (AJ581999)	99.71
EB252	*Pantoea rodasii* (JF295053)	98.81

Supplementary Table S2. Geographic proximity among sampling sites.

Geographic Locations	Latitude	Longitude	Altitude	Mean rainfall (mm) 2013	Mean temperature (°C) 2013	pH	Organic matter (g/kg)	Available phosphate (mg/kg)	K	Ca	Mg	Electrical condictivity
									($cmol^+$/kg)			
**A**	36° 51' 55.73" N	126° 26' 2.90" E	222.80m	94.4	22.2	5.2	23.8	242.8	1.3	1.8	0.6	0.6
**B**	38° 4' 29.16" N	127° 28' 12.73" E	448.74 m	23.3	22.8	5.4	42.9	436	0.6	4.7	1.6	0.4
**C**	36° 22' 0.35" N	127° 33' 23.73" E	206.74 m	162.6	23.9	6.3	23.1	289.9	0.7	7.1	1.9	0.3
**D**	36° 59' 26.80" N	129° 23' 36.28" E	250.72 m	146.1	19.3	6	20.3	168.7	3.4	4.8	1.8	0
**E**	36° 24' 44.35" N	127° 8' 24.97" E	389.30 m	151.7	23.1	5.7	19.8	309.3	0.5	4.1	1	0.6
**F**	37° 14' 3.19" N	128° 5' 47.90" E	501.92 m	199.2	23.9	6.3	25.5	483.7	0.7	6.9	2	0.6
**G**	37° 35' 42.43" N	128° 22' 28.41" E	615.98 m	36	21.5	5.4	27.9	570.9	0.8	4.1	1	0.7
**H**	37° 22' 23.82" N	128° 15' 57.84" E	509.00 m	158.2	22.7	5.5	24.8	506.1	1.2	5	1.3	0.6
**I**	37° 31' 1.93" N	128° 55' 38.31" E	562.09 m	112.6	19	5.9	114	721.6	4.9	7.5	1.1	0.7
**J**	36° 59' 5.25" N	127° 38' 40.13" E	400.11 m	229.9	24.6	5.8	17.9	374.8	0.4	3.6	1	0.4
**K**	36° 58' 16.11" N	128° 55' 16.06" E	557.54 m	160	20.3	5.6	18.3	254.7	0.5	5	2	0.1
**L**	37° 50' 17.47" N	128° 5' 5.26" E	577.15 m	84.6	23.3	5.6	22.7	450	0.7	3.9	1.1	0.5
**M**	35° 23' 2.00" N	127° 55' 15.86" E	256.85 m	99.5	23.1	4.5	23.5	17	0.9	2.9	0.6	0.3
**N**	37° 35' 34.97" N	127° 21' 41.70" E	271.34 m	87.7	23.7	5.9	24.6	320.2	0.5	5	1.1	0.6
**O**	36° 34' 5.94" N	128° 8' 40.93" E	217.53 m	125.7	22.9	7.7	22.1	232.3	0.7	6.1	2.1	0.7
**P**	37° 23' 45.24" N	127° 31' 45.16" E	308.04 m	115.1	23.3	5.8	20.6	300.3	0.4	3.9	1.1	0.7
**Q**	35° 59' 32.19" N	128° 42' 42.43" E	553.50 m	63.1	24.3	5.6	26.3	551	0.6	5.3	1.5	0.7
**R**	35° 35' 55.03" N	127° 38' 56.82" E	561.35 m	50.9	22.7	5.7	28.3	269.9	0.5	3.9	0.8	0.5
**S**	35° 17' 36.97" N	127° 21' 21.15" E	216.06 m	116.5	22	5.8	33.5	191.2	0.5	5	1.4	0.4
**T**	35° 48' 2.35" N	127° 18' 46.81" E	553.38 m	60.2	21.7	5.6	28.6	463.7	0.7	3.9	1	0.4
**U**	35° 33' 59.93" N	126° 54' 53.71" E	261.05 m	49.6	23.5	5.3	30.3	193.2	1	5.8	1.9	0.2
**V**	33° 23' 29.13" N	126° 19' 57.83" E	235.45 m	160.5	21.7	5.3	49.7	599.8	1.2	4.6	1.8	1.7
**W**	33° 19' 56.85" N	126° 22' 36.96" E	506.25 m	156	22.2	5.2	54.8	302	1.1	4.4	1.5	1.2
**X**	34° 59' 4.86" N	126° 29' 45.84" E	400.56 m	84.4	22	5.6	17	388	0.9	5.6	1.7	1.2

Supplementary Table S3. Different potential properties of bacterial endophytes isolated from MCG. '+' indicating the positive and '–' indicating the negative results for phosphate solubilization, production of siderophore, IAA-like indole derivatives and HCN, and ß-glucosidase activity.

Isolate no.	Siderophore production	Phosphate solubilization	IAA-like indole derivatives	HCN production	ß-glucosidase activity
EB1	-	-	+	-	+
EB2	-	-	-	-	+
EB3	+	+	-	-	-
EB4	+	+	+	-	-
EB5	-	-	+	-	+
EB6	+	+	+	-	-
EB7	-	-	-	-	+
EB8	+	+	+	-	+
EB9	+	+	+	-	+
EB10	-	-	-	-	+
EB11	-	-	-	-	-
EB12	+	-	+	-	+
EB13	-	-	-	-	-
EB14	-	+	-	-	-
EB15	-	-	-	-	+
EB16	-	-	-	-	-
EB17	+	-	-	-	-
EB18	+	+	+	-	+
EB19	+	-	+	-	+
EB20	+	+	-	-	-
EB21	+	+	+	+	-
EB22	+	-	-	-	-
EB23	+	+	-	-	-
EB24	+	+	-	-	-
EB25	+	-	+	-	+

Isolate no.	Siderophore production	Phosphate solubilization	IAA-like indole derivatives	HCN production	ß-glucosidase activity
EB26	+	+	+	-	-
EB27	+	-	+	-	+
EB28	+	+	+	-	-
EB29	+	+	+	-	-
EB30	-	-	+	-	+
EB31	-	-	+	-	+
EB32	+	+	-	-	-
EB33	+	+	+	-	-
EB34	+	+	+	+	-
EB35	+	+	-	-	-
EB36	-	-	-	-	-
EB37	+	+	+	+	-
EB38	+	+	+	-	-
EB39	+	-	-	-	-
EB40	+	+	+	-	+
EB41	-	-	+	-	+
EB42	-	+	+	-	+
EB43	+	-	-	-	-
EB44	-	-	-	-	+
EB45	+	-	-	-	-
EB46	+	+	-	+	-
EB47	-	-	+	-	+
EB48	-	+	-	-	-
EB49	+	+	-	+	-
EB50	+	+	+	-	-

Isolate no.	Siderophore production	Phosphate solubilization	IAA-like indole derivatives	HCN production	ß-glucosidase activity
EB51	+	-	+	+	-
EB52	+	-	-	-	+
EB53	+	+	+	-	-
EB54	+	+	+	-	-
EB55	-	-	+	-	-
EB56	-	-	+	-	+
EB57	+	-	+	-	+
EB58	+	-	+	-	-
EB59	-	+	+	-	+
EB60	+	-	-	-	-
EB61	+	-	-	-	-
EB62	+	-	+	-	-
EB63	+	-	+	-	+
EB64	+	+	+	-	-
EB65	+	-	+	-	+
EB66	+	-	+	+	-
EB67	+	-	+	-	+
EB68	-	-	+	-	+
EB69	+	-	+	-	+
EB70	-	-	-	-	-
EB71	+	-	+	-	-
EB72	+	-	-	-	+
EB73	-	-	-	-	-
EB74	+	+	-	-	-
EB75	+	+	+	-	-

Continued

Isolate no.	Siderophore production	Phosphate solubilization	IAA-like indole derivatives	HCN production	ß-glucosidase activity
EB76	-	-	+	-	-
EB77	+	-	-	-	-
EB78	+	-	-	-	+
EB79	+	+	+	-	-
EB80	+	-	-	-	-
EB81	-	-	+	-	-
EB82	+	-	+	+	-
EB83	+	+	+	-	-
EB84	+	+	+	-	-
EB85	+	-	+	-	-
EB86	+	+	+	-	-
EB87	+	+	+	+	-
EB88	+	+	-	-	-
EB89	+	+	+	-	+
EB90	+	+	-	+	-
EB91	-	-	+	-	+
EB92	-	-	+	-	+
EB93	+	-	+	-	+
EB94	-	+	+	-	-
EB95	+	-	+	-	-
EB96	-	-	+	-	+
EB97	-	-	-	-	+
EB98	-	-	-	-	+
EB99	-	+	+	-	+
EB100	-	+	-	+	-

Isolate no.	Siderophore production	Phosphate solubilization	IAA-like indole derivatives	HCN production	ß-glucosidase activity
EB101	+	-	-	-	+
EB102	+	-	-	-	+
EB103	+	-	+	-	-
EB104	+	+	+	-	-
EB105	-	-	+	-	+
EB106	-	+	+	-	+
EB107	+	+	+	-	-
EB108	+	+	+	-	-
EB109	+	-	+	-	+
EB110	+	-	+	-	+
EB111	+	-	+	-	+
EB112	-	-	+	-	-
EB113	-	-	+	-	+
EB114	+	+	+	-	+
EB115	-	-	+	-	+
EB116	-	-	+	-	+
EB117	-	-	+	-	+
EB118	+	+	+	-	-
EB119	-	-	-	-	+
EB120	+	-	+	-	+
EB121	+	+	-	-	-
EB122	+	-	-	-	+
EB123	+	+	-	-	-
EB124	-	-	+	-	+
EB125	-	-	-	-	+

Isolate no.	Siderophore production	Phosphate solubilization	IAA-like indole derivatives	HCN production	ß-glucosidase activity
EB126	-	-	-	-	-
EB127	+	-	-	-	+
EB128	+	-	+	-	+
EB129	-	-	+	-	-
EB130	+	+	+	+	-
EB131	+	+	-	-	-
EB132	+	-	-	+	-
EB133	+	+	+	-	-
EB134	-	-	+	-	-
EB135	+	+	+	-	-
EB136	-	+	+	-	+
EB137	+	+	-	-	-
EB138	+	+	-	-	-
EB139	+	-	+	-	-
EB140	+	-	+	-	+
EB141	+	+	+	-	+
EB142	+	-	+	+	-
EB143	+	+	+	-	-
EB144	+	-	+	-	-
EB145	+	-	+	-	-
EB146	+	+	-	-	-
EB147	+	+	+	-	-
EB148	-	+	-	-	-
EB149	+	+	+	-	+
EB150	+	-	-	-	+

Continued

Isolate no.	Siderophore production	Phosphate solubilization	IAA-like indole derivatives	HCN production	ß-glucosidase activity
EB151	+	-	+	+	-
EB152	+	+	+	+	-
EB153	-	-	-	-	-
EB154	+	+	+	-	-
EB155	-	-	-	-	-
EB156	-	-	-	-	-
EB157	-	+	-	-	+
EB158	-	-	+	-	+
EB159	+	+	+	-	-
EB160	+	-	+	-	-
EB161	+	-	+	-	+
EB162	+	+	+	-	-
EB163	+	+	+	-	-
EB164	+	-	+	+	-
EB165	-	+	+	+	-
EB166	+	+	-	+	-
EB167	+	+	+	+	-
EB168	+	+	+	-	+
EB169	+	+	+	+	+
EB170	+	+	+	-	-
EB171	+	-	-	-	-
EB172	+	+	+	-	+
EB173	+	+	-	-	-
EB174	+	-	-	+	-
EB175	+	-	-	+	-
EB176	-	-	-	-	+
EB177	+	+	+	-	-
EB178	+	+	+	-	-
EB179	+	-	+	+	-
EB180	+	+	+	-	-
EB181	+	+	-	-	-
EB182	+	+	-	+	-
EB183	-	+	+	-	-
EB184	-	+	-	-	-
EB185	-	-	+	-	-
EB186	+	+	-	-	-
EB187	-	-	-	+	-
EB188	+	-	-	-	-
EB189	+	-	-	-	-
EB190	+	-	-	+	-
EB191	+	-	+	-	-
EB192	+	-	+	-	-
EB193	+	+	+	-	+
EB194	+	-	+	-	-
EB195	+	-	+	-	+
EB196	+	-	+	-	+
EB197	+	-	+	-	+
EB198	+	+	-	-	-
EB199	+	-	+	-	-
EB200	+	+	+	-	-
EB201	+	-	+	-	+
EB202	+	+	+	-	-
EB203	-	-	+	-	-
EB204	-	-	+	-	+
EB205	+	+	+	-	-
EB206	+	-	+	-	-
EB207	+	-	+	-	+
EB208	+	+	-	+	-
EB209	+	+	-	-	-
EB210	-	+	-	+	-
EB211	+	+	-	+	-
EB212	+	+	+	-	+
EB213	+	-	+	-	+
EB214	+	-	+	-	+
EB215	+	-	+	-	-
EB216	+	+	+	-	+
EB217	+	-	+	-	+
EB218	+	-	+	-	-
EB219	+	+	+	+	-
EB220	+	+	+	-	+
EB221	+	+	+	-	+
EB222	+	-	+	-	+
EB223	+	+	+	+	-
EB224	-	-	+	-	-
EB225	-	-	-	-	+

Continued

Isolate no.	Siderophore production	Phosphate solubilization	IAA-like indole derivatives	HCN production	ß-glucosidase activity	Isolate no.	Siderophore production	Phosphate solubilization	IAA-like indole derivatives	HCN production	ß-glucosidase activity	Isolate no.	Siderophore production	Phosphate solubilization	IAA-like indole derivatives	HCN production	ß-glucosidase activity
EB226	+	+	+	-	-	EB235	+	+	-	-	-	EB244	+	+	+	-	-
EB227	+	-	+	-	-	EB236	+	+	-	-	-	EB245	+	+	+	-	-
EB228	+	-	+	-	+	EB237	+	+	+	-	-	EB246	+	-	+	-	+
EB229	+	-	+	-	-	EB238	+	+	+	-	+	EB247	+	+	+	-	-
EB230	+	-	+	-	+	EB239	+	+	+	-	+	EB248	+	-	-	-	+
EB231	-	-	+	-	-	EB240	+	+	+	-	+	EB249	+	-	-	-	-
EB232	-	-	+	-	+	EB241	+	-	-	-	-	EB250	-	+	-	-	+
EB233	+	+	+	-	-	EB242	+	-	+	-	+	EB251	+	+	-	-	-
EB234	+	+	+	-	+	EB243	+	-	+	-	-	EB252	+	+	-	-	+
												**Total**	**185**	**118**	**168**	**32**	**98**
												**(%)**	**73.41**	**46.82**	**66.66**	**12.69**	**38.88**
